# Precise integrin-targeting near-infrared imaging-guided surgical method increases surgical qualification of peritoneal carcinomatosis from gastric cancer in mice

**DOI:** 10.18632/oncotarget.14058

**Published:** 2016-12-21

**Authors:** Haidong Cheng, Chongwei Chi, Wenting Shang, Sha Rengaowa, Jianxin Cui, Jinzuo Ye, Shixin Jiang, Yamin Mao, Caoting Zeng, Huiping Huo, Lin Chen, Jie Tian

**Affiliations:** ^1^ Department of General Surgery, The Chinese PLA General Hospital, Beijing, 100853, China; ^2^ Department of General Surgery, The First Affiliated Hospital of Inner Mongolia Medical University, Hohhot, 010059, China; ^3^ Key Laboratory of Molecular Imaging of the Chinese Academy of Sciences, Institute of Automation, Chinese Academy of Sciences, Beijing, 100190, China; ^4^ Department of Basic Medical Science, Inner Mongolia Medical University, Hohhot, 010059, China; ^5^ Department of Hepatobiliary Surgery, Zhujiang Hospital, Southern Medical University, Guangzhou, 510280, China; ^6^ Department of Ultrasound, General Hospital of the People's Liberation Army, Beijing, 100853, China

**Keywords:** gastric cancer, fluorescence molecular imaging, peritoneal carcinomatosis, integrin

## Abstract

Peritoneal carcinomatosis from gastric cancer represents a common recurrent gastric cancer that seriously affects the survival, prognosis, and quality of life of patients at its advanced stage. In recent years, complete cytoreduction surgery in combination with hyperthermic intraperitoneal chemotherapy has been demonstrated to improve the survival and prognosis of patients with malignant tumors including peritoneal carcinomatosis from gastric cancer. Establishing viable methods of accurately assessing the tumor burden in patients with peritoneal carcinoma and correctly selecting suitable patients in order to improve cytoreduction surgical outcomes and reduce the risk of postoperative complications has become a challenge in the field of peritoneal carcinoma research. Here, we investigated peritoneal carcinomatosis from gastric cancer in a mouse model by using our self-developed surgical navigation system that combines optical molecular imaging with an integrin-targeting Arg-Gly-Asp-indocyanine green (RGD-ICG) molecular probe. The results showed that our diagnostic method could achieve a sensitivity and specificity of up to 93.93% and 100%, respectively, with a diagnostic index (DI) of 193.93% and diagnostic accuracy rate of 93.93%.Furthermore, the minimum tumor diameter measured during the surgery was 1.8 mm and the operative time was shortened by 3.26-fold when compared with the conventionally-treated control group. Therefore, our surgical navigation system that combines optical molecular imaging with an RGD-ICG molecular probe, could improve the diagnostic accuracy rate for peritoneal carcinomatosis from gastric cancer, shorten the operative time, and improve the quality of the cytoreduction surgery for peritoneal carcinomatosis from gastric cancer, thus providing a solid foundation for its future clinical development and application.

## INTRODUCTION

Gastric cancer is a common malignant tumor that accounts for approximately 10% of patients with new-onset tumors and 12% of cancer-related deaths, ranking it fourth among malignant tumor occurrences and second for mortality rate. Although the therapeutic approaches and diagnostic methods for neoplastic diseases have significantly improved in recent years, the 5-year survival rate of patients with gastric cancer remainsbelow 30% [1]. East Asian countries including Japan, South Korea, and China in particular have a high incidence rate of gastric cancer that accounts for approximately 60% of the worldwide incidence of gastric cancer. Among these, China accounts for 40%,primarily comprising patients with advanced cancer [2–4] such asperitoneal carcinomatosis from gastric cancer, whichrepresents a common recurrence and metastasis of gastric cancer. Despite radical treatment for advanced gastric cancer, 50% of patients exhibitgastric cancer recurrence or metastasis. Furthermore, nearly 12% of patients were found with peritoneal carcinomatosis from gastric cancer during the treatment and diagnosed with advanced gastric cancer, which has seriously affected the survival, prognosis, and quality of life of these patients [5–7].

Features of peritoneal carcinomatosis from gastric cancer include positive exfoliated free cells (positive cytology; CY+) and visible disseminated peritoneal nodules (peritoneal metastasis; P+) [8], of which the visible disseminated peritoneal carcinomatosis nodules of gastric cancer, ovarian cancer, and colorectal cancer, as well as primary peritoneal mesothelioma, are commonly termed the peritoneal carcinoma [9–11]. Notably, it has been long believed that peritoneal carcinoma owing to peritoneal carcinomatosis from gastric cancer represents a non-curable advanced cancer with a survival prognosis of not more than 6 months [12–14]. Some clinical studies have evaluated the therapeutic efficacy of various approaches including systemic chemotherapy, intraperitoneal chemotherapy, and local radiotherapy and chemotherapy for this disease;however, significant determination of efficacy has not been established. Patients undergoing palliative surgery, systemic chemotherapy, and nutrition support therapy retainthe survival prognosis of not more than one year [15, 16]. However, researchers are still actively exploring potential therapeutic methods for malignant peritoneal carcinoma including for peritoneal carcinomatosis from gastric cancer.

In recent years, the combination of complete cytoreduction surgery and hyperthermic intraperitoneal chemotherapy has been shown to significantly improve the survival and prognosis of patients with malignant tumors, such as peritoneal carcinomatosis from colon or gastric cancer, peritoneal mucinous tumors, and ovarian cancer [17, 18]. However, at present it is still not possible to recommend the combination of cytoreduction surgery and hyperthermic intraperitoneal chemotherapy as a clinical treatment because of several remaining concerns [19]. First, the selection of suitable patients is difficult. In addition to the establishing the type of affected tissues, the evaluation and selection of suitable patients mainly relyupon the peritoneal carcinoma index (PCI) as a key indicator,which is closely associated with the size and location of the tumor; however, it is difficult to effectively obtain an accurate PCI in clinical practice.Second, an accurate intraoperative assessment of surgical outcomes is also difficult. The completeness of cytoreduction (CCR) score is significantly associated with postoperative prognosis. However, it is difficult to achieve CCR0-1 in the clinical surgery or precise resection that results in no residual lesion or residual lesions with the greatest diameter less than 2.5 mm, thereby leading to poor survival.The third concern comprises associated severe complications.Studies have shown that cytoreduction surgery and hyperthermic intraperitoneal chemotherapy result in morbidity and mortality rates of 30% and 3%, respectively [20]. The high incidence rate of complications owing to cytoreduction surgery and hyperthermic intraperitoneal chemotherapy is closely associated with several factors, such as diagnostic accuracy rate, operative time, and the learning curve of the treating physician [21]. Accordingly, methods of accurately assessing the tumor burden in patients with peritoneal carcinoma and selecting suitable patients to enable the timely feedback of surgical outcomes and reduce the risk of postoperative complications are becoming areas of focused peritoneal carcinoma research.

Preoperative imaging modalities, such as ultrasound, computerized tomography , MRI, and positron emission tomography , have been widely used in preoperative diagnoses and have become an important preoperative assessment approach for peritoneal carcinomatosis from gastric cancer [22]. However, the existing medical imaging methods still have difficultyin detecting peritoneal carcinomatosis from gastric cancer with lesion sizes less than 5 mm, which has impeded the accurate selection of suitable patients [23]. The current diagnosis and treatment for peritoneal carcinomatosis from gastric cancer mainly rely on laparotomy or laparoscopy that concurrently relies on palpation and visualization by surgeons. As an alternative, optical molecular imaging has been shown in recent years to achieve an accurate diagnosis and treatment for neoplastic diseases [24] with multiple advantages for peritoneal carcinomatosis from gastric cancer. Specifically, optical molecular imaging(1) exhibits a high diagnostic accuracy that improves the accuracy of tumor staging; (2) can detect small lesions; and (3) can precisely determine the tumor boundary.Furthermore, optical molecular imaging (4) functions as a real-time and rapid imaging modality and (5) is considered an objective imaging technique that (6) is free of hazardous radiation and is inexpensive and easy to operate. (7) Finally,this imaging method enables precise surgery for peritoneal carcinoma.

Recently van Damet al.demonstrated the first utilization of the combination of a folate receptor-α-fluorescein isothiocyanate molecular probe with optical imaging devices in clinical applications for ovarian cancer [25]. The results showed that fluorescence molecular imaging could detect more tumor lesions and improve tumor staging compared with conventional observation and assessment methodologies. In addition, Almerie et al. performed a clinical study of 5-aminolevulinic acid (5-ALA) for fluorescence imaging of peritoneal carcinoma, which indicated that thismethod couldachieve up to 83–100% and 95–100% sensitivity and specificity, respectively, representing an improvement of the diagnostic accuracy rate by 10–20% compared with the conventional visualization and assessment method [26]. Therefore, the combination of a synthetic molecular probe specifically targeting the tumor tissues along with optical imaging devices would likely improve the clinical outcomes of peritoneal carcinoma. In particular, integrins have been shown to be widely expressed in tumor tissues and blood vessels of gastric cancer compared with normal tissues, and have accordingly become new therapeutic targets for gastric cancer [27]. Notably, synthetic Arg-Gly-Asp (RGD) peptides targeting integrins have entered Phase I of clinical trials [28]. In the current study, we designed and synthesized an RGD-ICG molecular probe targeting integrins to achieve near-infrared imaging and image-guided precision surgery by using our self-developed optical molecular imaging device.The successful outcomesof this strategy provide a good theoretical foundation for the future clinical application of optical molecular imaging during surgical treatment for peritoneal carcinomatosis from gastric cancer.

## RESULTS

### Expression of integrin α_v_β_3_ in gastric cancer cell lines

**Figure 1:**
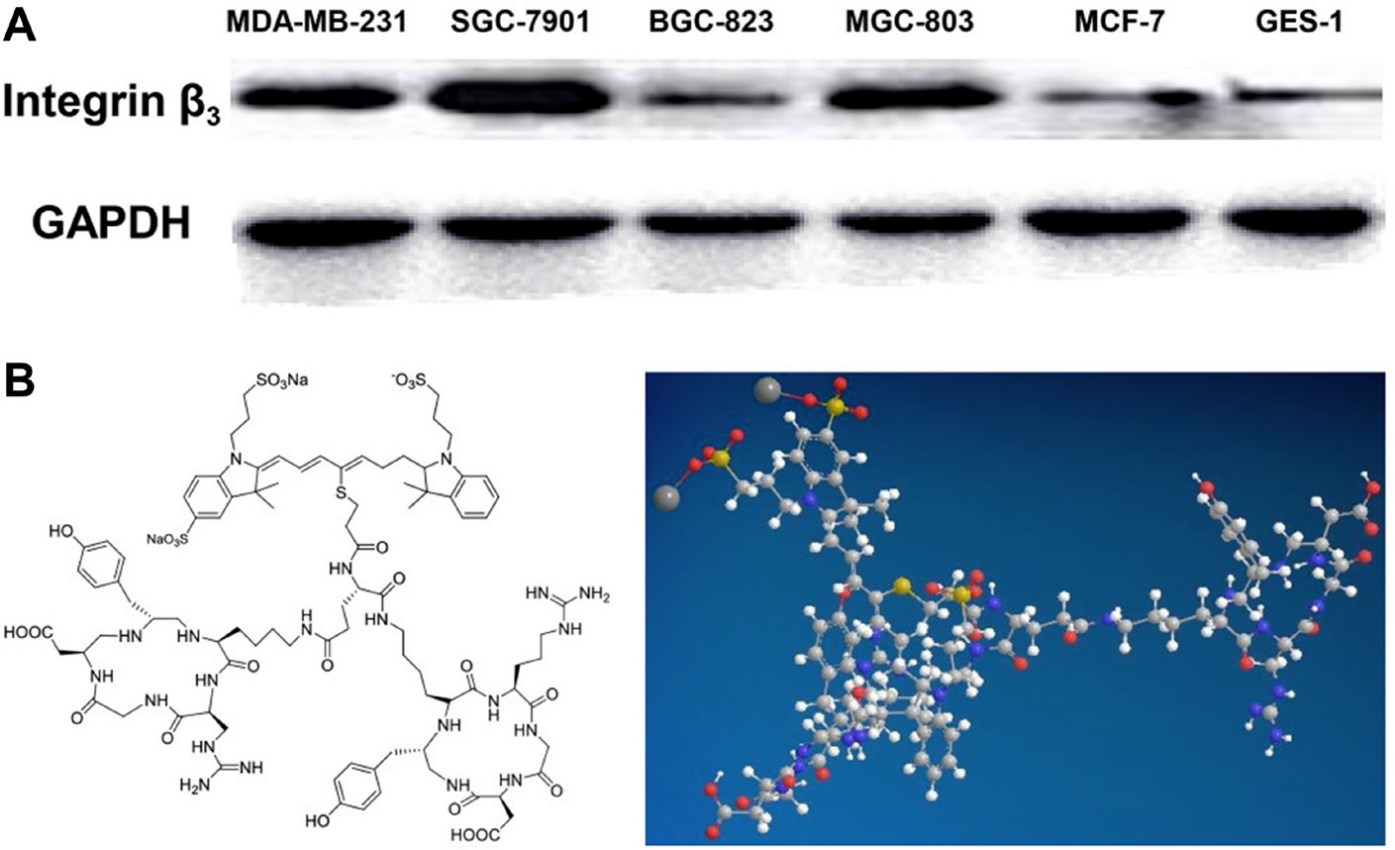
Expression of β3 in corresponding cell lines and the structure of the probe RGD-ICG (**A**) Integrin subunit β3 has a higher expression level in the gastric cancer cell line SGC-7901, compared with the breast cancer cell line MDA-MB-231, which has a high expression level of integrin αvβ3, by using GAPDH as the internal reference. GES-1 has a expression level of integrin subunit β3 similar to the breast cancer cell line MCF-7, which has a low expression of integrin αvβ3; (**B**) the chemistry and 3D structure of the probe RGD-ICG.

In this study, the expression of the integrin α_v_β_3_ subunit β3 in gastric cancer cell lines SGC-7901, BGC-823, and MGC-803; breast cancer cell lines MDA-MB-231 (high expression) and MCF-7 (low expression); as well as in the normal gastric epithelial cell line GES-1 was determined by western-blot. As shown in Figure 1, the integrin subunit, β3exhibits higher expression in the gastric cancer cell line SGC-7901 compared with the breast cancer cell line MDA-MB-231 as determined by usingGAPDH as the internal reference protein whereas in GES-1, its expression level is similar to that of the breast cancer cell line MCF-7, which shows low expression of integrin α_v_β_3_.

### *In vitro* cytological assessment of the molecular probes

**Figure 2:**
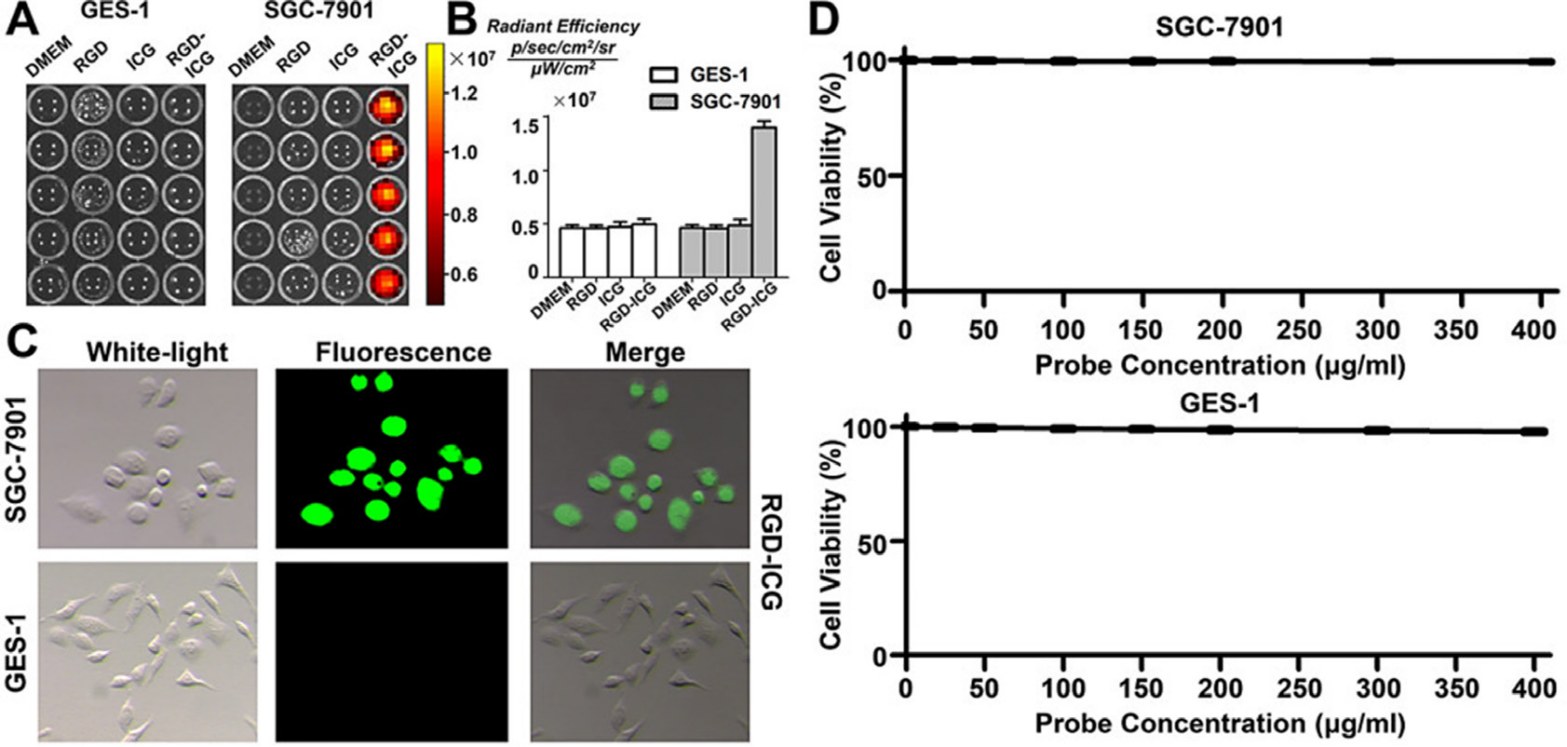
The targeting ability and toxicity of RGD-ICG in cell lines (**A**) The fluorescence signals in SGC-7901 and GES-1 cells after incubated with RGD, ICG, and RGD-ICG; (**B**) Quantitative analysis of fluorescence signals in each group. The differences in fluorescence signal intensity between SGC-7901+RGD-ICG with SGC-7901+ICG, SGC-7901+RGD, SGC-7901, and GES-1+RGD-ICG were statistically significant; (**C)** The targeting ability of RGD-ICG in SGC-7901 and GES-1 under a fluorescence microscope; (**D)** The toxicity of RGD-ICG in SGC-7901 and GES-1.

To validate the ability of the probe to target gastric cancer cells in the cellular environment, we utilized the gastric cancer cell line SGC-7901 with high expression of integrin α_v_β_3_ as the experimental group whereas the normal gastric epithelial cell line GES-1, with low expression of integrin α_v_β_3_, was used as the negative control group. The cells of these two groups were treated for 30 min with Dulbecco's modified Eagle's medium (DMEM), free RGD, pure ICG, and RGD-ICG, followed by detection of the fluorescence signal using the IVIS imaging system. As shown in Figure 2A, the GES-1 cell line, which has low expression of integrin α_v_β_3_, produced a lower fluorescence signal in each group, whereas the tumor cells of the gastric cancer cell line SGC-7901 specifically recognized and bound to the RGD sequence in order to initiate endocytosis, resulting from the high level of integrin expression in this cell line. Although free RGDs were theoretically capable of entering these cells, no fluorescence signal would be produced as the RGDwas not connected to the fluorescent dye. Subsequently, the normal tissues and cells would not uptake pure ICG added to the medium;thus,no fluorescence signal would be generated after repeated washing. Figure 2B further indicates that the quantitative targeting ability of the RGD-ICG probes exhibited a marked difference between the tested cell lines.Additionally, images of the two cell types captured under a fluorescence microscope clearly revealed the targeting ability of the RGD-ICG molecular probes toward the gastric tumor cells (Figure 2C).

In order to determine the toxicity of the probe, we evaluated the effect of different concentrations of RGD-ICG solution on cell viability by using the MTT assay. As shown in Figure 2D, the viability of the cells remained unaffected by increasing concentration of RGD-ICG in the cell culture medium. Gastric cancer SGC-7901 cells and normal gastric epithelial GES-1 cells retained cell viabilities above 90% even when the probe concentration increased to 400 mg/ml. This result indicated that the RGD-ICG fluorescence molecular probe was non-toxic to the tested tissues and cells and exhibited good histocompatibility, thus supporting its likely utility in future applications.

### *In vivo* metabolic profiling of the molecular probes

**Figure 3:**
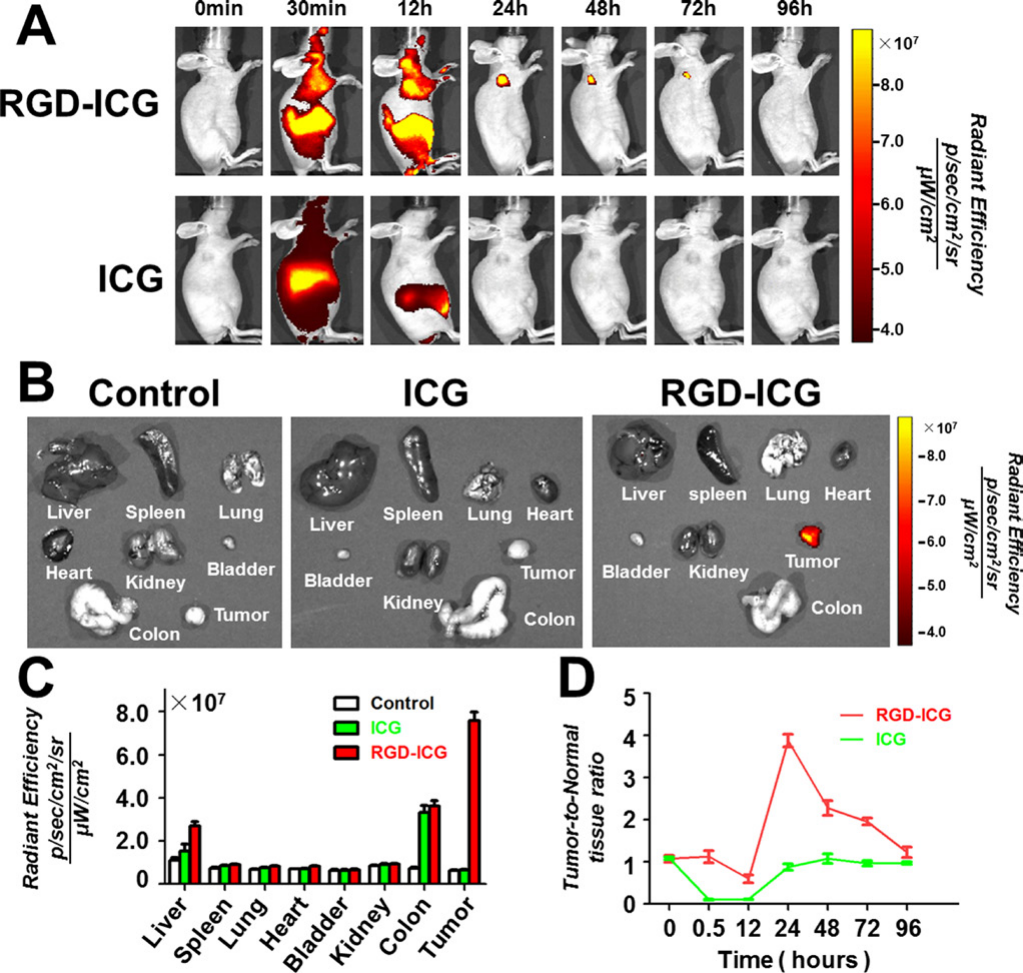
In vivo metabolic profiling of the molecular probes (**A)** RGD-ICG administered via intravenous tail injection gradually accumulates in the subcutaneous tumor with retention time up to 72 hours, whereas the pure ICG is digested and excreted from the body within 24 hours as the tumor tissue cannot uptake pure ICG; (**B)** The fluorescence distribution in tumor tissues and main thoracic-abdominal organs in nude mice 24 hours after intravenous tail injection of RGD-ICG and ICG. Of which, nude mice in the control group, ICG group, and RGD-ICG group were administered with pure PBS, pure ICG, and RGD-ICG molecular probes, respectively, via intravenous tail injection. The data placed under the same scale of fluorescence intensity show that the fluorescence signals in various tissues and organs in the control and ICG groups were too low for imaging. In contrast, RGD-ICG retained in the tumor tissue generated a higher fluorescence intensity for the imaging; (**C)** Quantitative analysis of fluorescence signals in the above-mentioned tissues. (**D)** The tumor-to-normal tissue ratio reached up to about 4 and 1 at 24 hours after injection of RGD-ICG and ICG, respectively.

To verify the *in vivo* targeting ability of the molecular probes and the optimum time point for imaging, we compared and analyzed the fluorescence imaging results at different time points at the same scale of fluorescence signal. It can be seen from Figure 3A that the whole body of nude mice bearing subcutaneous gastric cancer tumors emitted fluorescence signal after receiving intravenous tail injection of the RGD-ICG probe. After 30 min, the probe began to accumulate in the tumors and the liver. Probes in other part of the body were digested and excreted from the body via the digestive tract within 24 h after probe injection, which significantly reduced the background noise while the probes accumulating in tumors retained a strong fluorescence signal intensity. Subsequently, the fluorescence signal attenuated to a lower intensity after 72 h of continual monitoring. In contrast, ICG was unable to reveal the gastric cancer tissues under imaging as the tumor tissue could not uptake pure ICG. Notably, these results were consistent with the conclusions of the cytology experiment.

To determine the distribution of probes in the organs of mice at the optimum time point (24 h), we sacrificed the nude mice 24 h after probe injection to obtain the organs for fluorescence signal detection (Figure 3B). Mice in the control group injected with PBS buffer and in the ICG group exhibited lower 24-h post-injection signal intensity, whereas the tumors in mice that received the RGD-ICG probe injection exhibited apparently higher signal intensity. The results of *in vivo* imaging were next further validated by *in vitro* experiments. Figure 3C shows the signal intensity in each organ whereas Figure 3D shows the tumor-to-normal tissue ratio of the fluorescence signal at different time points. From these data, it can be seen that the signal-to-noise ratio peaked at 24 h.

### Optical imaging system-guided precision surgery for gastric cancer

**Figure 4:**
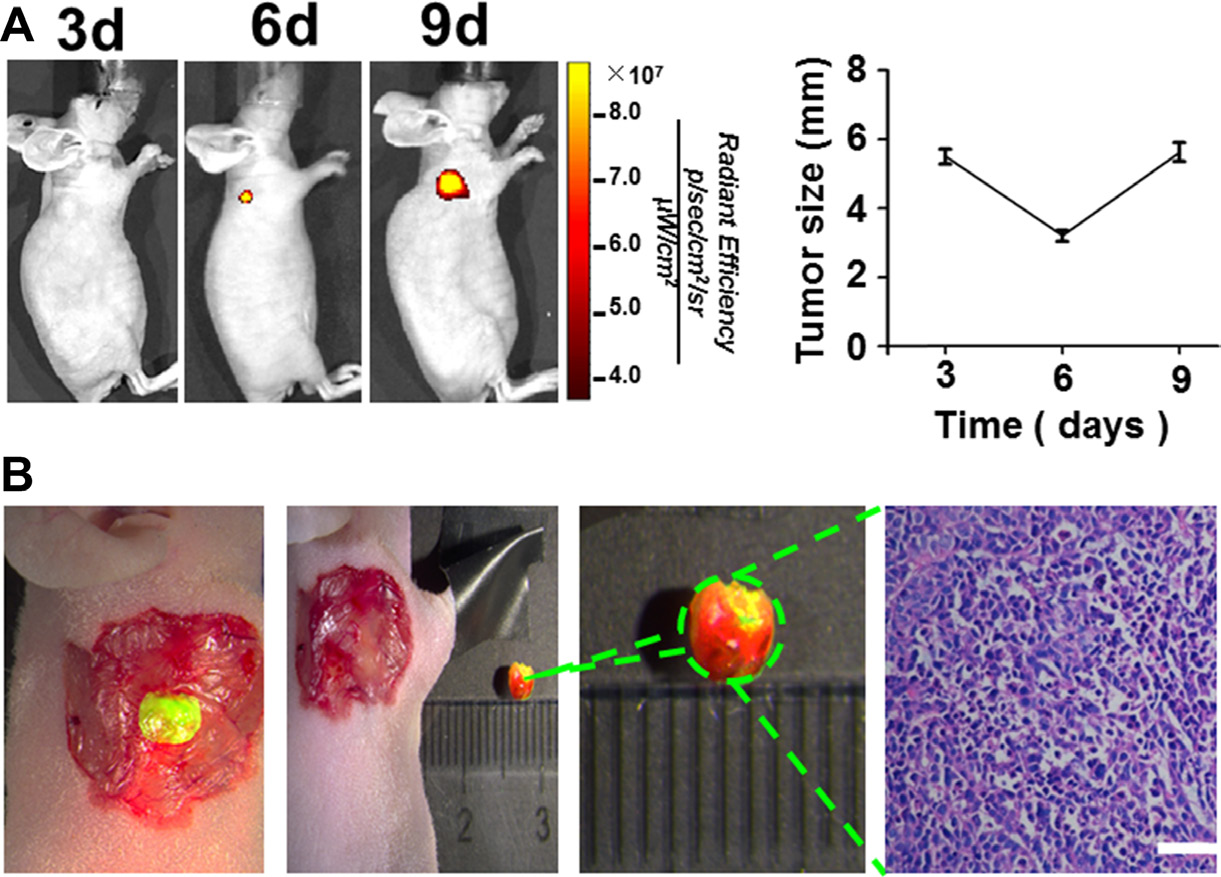
The imaging time of subcutaneous tumors of gastric cancer cell line SGC-7901 and the fluorescence imaging guided surgery (**A**) The representative nude mice shows that the gastric carcinoma cell line, SGC-7901 can be subjected to imaging at 6th day after subcutaneous implantation; and the tumor tissue shrunk at the beginning to the minimum size at about 6th day before gradually enlarged. scale bar = 50 μm; (**B**) the fluorescence imaging guided surgery in a subcutaneous gastric cancer nude mice model.

To verify the surgical outcomes of the RGD-ICG-based optical imaging system for gastric cancer, we conducted a surgery navigated by this system for subcutaneous gastric cancer tumors. As shown in Figure 4A, a clear image of tumor tissues at the 6th day after implantation was obtained 24 h after injection of RGD-ICG. In contrast, tumor tissues at the 3rd day exhibited a poor signal-to-noise ratio for imaging compared with normal tissues. The tumors gradually shrank at the early stage of subcutaneous implantation with the gastric cancer cell line, SGC-7901, to a minimum size of approximately 3.4 ± 0.3 mm at the 6th day, but they gradually enlarged afterward. It could be seen that tumors of the gastric cancer cell line, SGC-7901, could be subjected to imaging 6 days after implantation.

We carried out the surgery on selected nude mice with subcutaneous tumors at the 6th day after implantation. The fluorescence detection was performed using our imaging system 24 h after intravenous tail injection of RGD-ICG,which detected the presence of fluorescence accumulation on the neck of the nude mice. Tissues with the greatest fluorescence signal intensity were excised and pathologically verified as cancerous tissue. Additionally, the fluorescence signal at the resection area disappeared after the surgery, which could thereby provide a timely feedback to the surgeon regarding the surgical outcome (Figure 4B).

**Figure 5:**
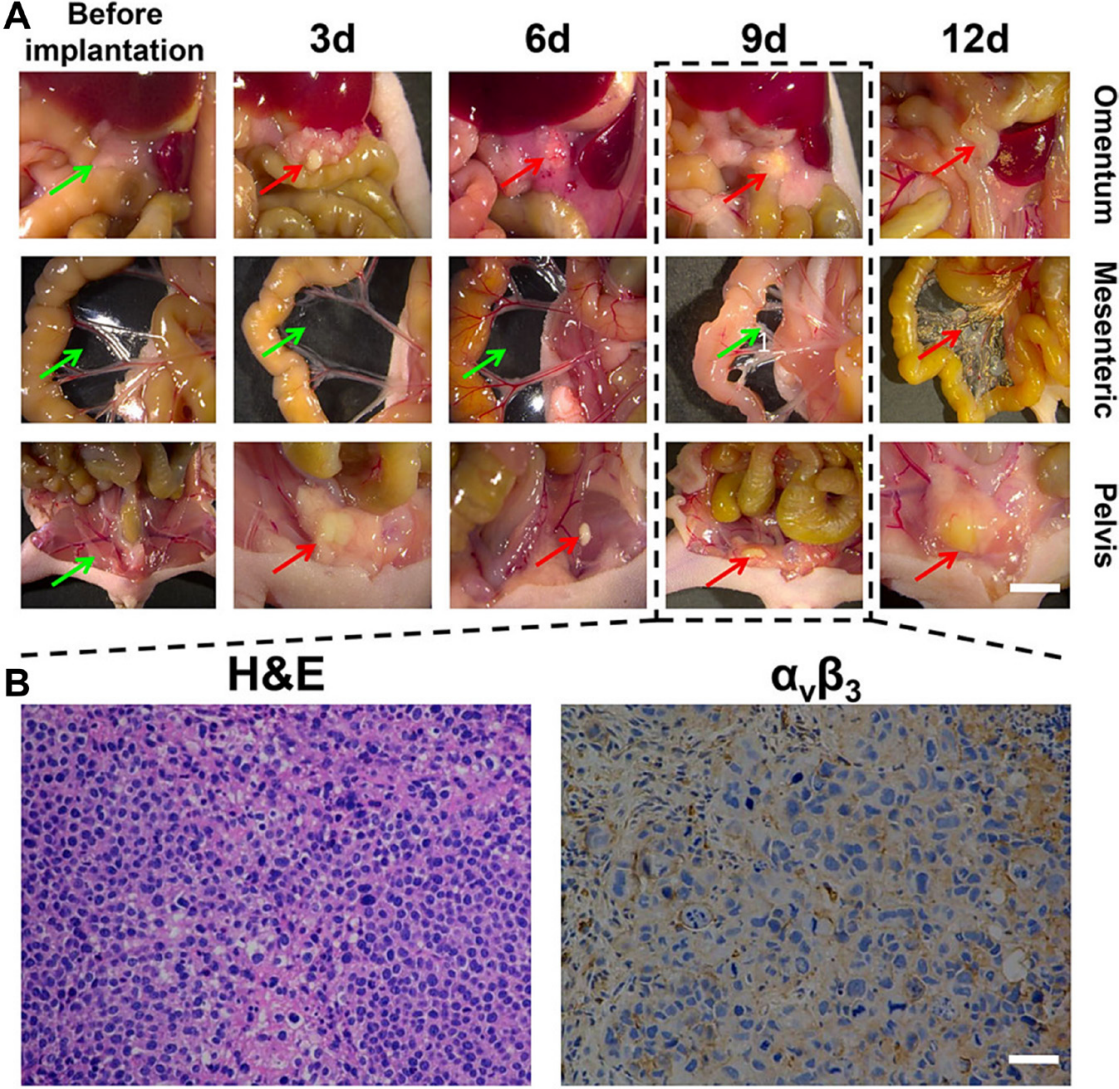
The dynamic and histological features of gastric cancer cell line SGC-7901 in peritoneal tumor formation (**A**) shows the morphological characteristics of tumors at different time points. The mesenteric tumor nodules at varying sizes were widely disseminated and can no longer be counted 12 days after implantation of tumor cells. Models at approximately the 9th day were selected, of which the green arrows indicate no pathological finding of tumor invasion ,while red arrows indicate cancerous tumor, Scale Bar = 5 mm; (**B**) shows the histological examination of three randomly selected tumor nodules from nude mice model at 9th day. It can be seen that there was a large number of abnormal cells and integrin α_v_β_3_ was significantly expressed in the tumor tissue. Scale Bar = 50 μm.

On the basis of the subcutaneous tumor research, we explored the potential of imaging and surgery for peritoneal carcinomatosis from gastric cancer. As shown in Figure 5A, we carried out laparotomy on nude mice prior to the injection of the gastric cancer cell line, SGC-7901, and at day 3, 6, 9, and 12 post-injection, in order to visualize the tumor characteristics. In the early stage after the intraperitoneal injection of tumor cells, the vast majority of individual cells dispersed in the intraperitoneal cavity whereas tumor cells aggregated into clusters could be seen in the omentum and pelvic cavity. Over time, the nodules shrank, but subsequently gradually enlarged and hardened. At the 12th day after tumor cell implantation, a large number of disseminated and merging mesenteric tumor nodules at different sizes appeared in the nude mice model alongside the growth of tumor cells in the intraperitoneal cavity. In addition, the tumor nodules in the omentum and pelvic cavity had enlarged. Therefore, we selected the nude mouse model at the 9th day after implantation for subsequent analysis owing to the isolated dispersion of tumor nodules and other normal nodules in the intraperitoneal cavity, which were easy to count. The gray and rigid tumor nodules were seen to be distributed on the surfaces of the organs and parietal peritoneum, which was consistent with the model of peritoneal carcinomatosis from gastric cancer. We then randomly selected three tumor tissue samples for further histological examination. As shown in Figure 5B, hematoxylin & eosin (H&E) staining revealed a large number of abnormal cells distributed within the tissue and a substantial expression of integrin α_v_β_3_ in the tumor tissue. Furthermore, as integrins represent highly expressed enzymes during angiogenesis, we also performed staining for CD31 and found that it was highly expressed, thereby confirming the specific binding of the RGD-ICG molecular probe to the peritoneal carcinomatosis, which provides the material foundation for tumor imaging.

**Figure 6:**
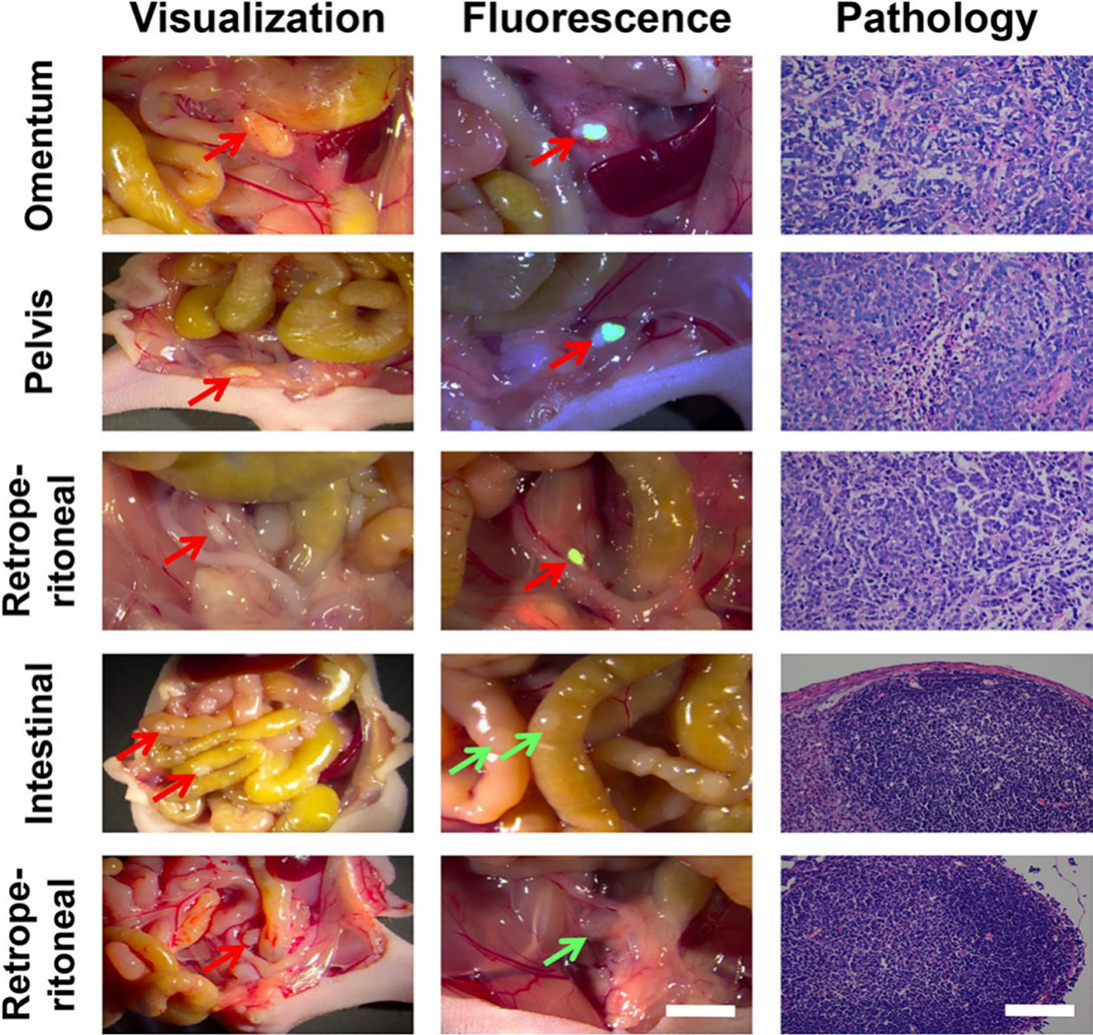
The application of RGD-ICG-based optical system in the cytoreduction surgery for peritoneal carcinomatosis from gastric cancer Both physicians in the conventional group were capable of accurately identifying tumor nodules in omentum, retroperitoneal, and pelvic cavity. Physicians in the fluorescence group accurately identified tumor nodules in the intraperitoneal cavity by using the navigation system, whereas the images of normal nodules were unable to be captured. The nature of all nodules found in the experiment has been pathologically confirmed. In the figure, red arrows represent identified positive lesions ,while green arrows represent negative nodules, *in vivo* scale bar = 5 mm, H&E scale bar = 50 μm.

To further improve the diagnostic performance, accuracy rate, and PCI accuracy rate of peritoneal carcinomatosis from gastric cancer as well as to facilitate the selection of suitable patients and reduce the risk of postoperative complications, we utilized the RGD-ICG molecular probes and optical imaging system to specifically differentiate tumor tissues from normal tissues during laparotomy. Figure 6 shows tumor lesions identified by both the conventional and fluorescence groups (red arrows) as well as lesions with no fluorescence detectable by imaging and which were pathologically confirmed as benign nodules (green arrows), indicating the specificity of molecular imaging during the surgery for peritoneal carcinomatosis from gastric cancer.

Tables 1 and 2 show the number of tumors identified and excised from the fluorescence- and conventional-detection groups, respectively. The RGD-ICG-based optical diagnostic system used in this study achieved a sensitivity of 93.93%, specificity of 100%, DI of 193.93%, and diagnostic accuracy rate of 93.93%, in the examination of peritoneal carcinomatosis from gastric cancer. The diagnostic accuracy rate of the conventional group was 76.04%.

**Table 1:** The laparotomy results of fluorescent group

Fluorescence	Histopathology		Total
	Positive	Negative	
Positive	31	0	31
Negative	2	69	71
Total	33	69	102

**Table 2:** The laparotomy results of conventional group

Visualization + Palpation	Histopathology		Total
	Positive	Negative	
Positive	29	23	52
Negative	0	44	44
Total	29	67	96

**Figure 7:**
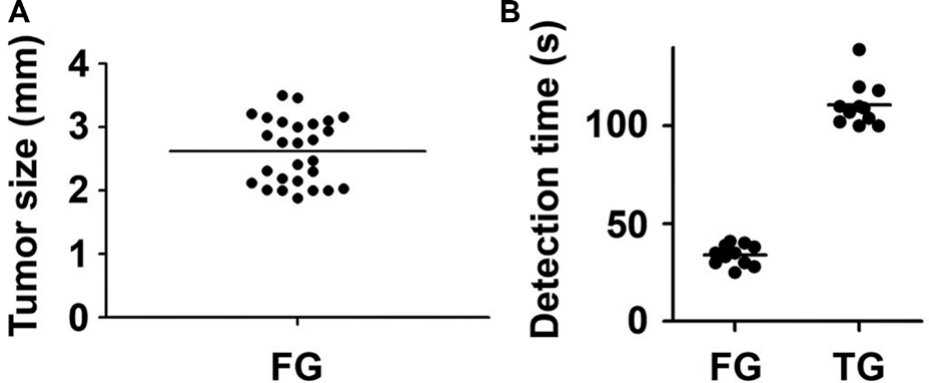
The tumor size and detection time of laparotomy performed by the fluorescence and the conventional groups (**A**) The size distribution of tumors excised by the fluorescence group; (**B**) The detection time of laparotomy performed by the fluorescence group was significantly shorter than that of the conventional group (*P < 0.05), FG: Fluorescence group, CG: Convention group.

In this study, the RGD-ICG-based optical diagnostic system could detect small tumor lesions. As shown in Figure 7A, the mean maximum tumor diameter was 2.6 ± 0.5 mm whereas the minimum tumor diameter was 1.8 mm, which might provide guidance to physicians for the precise removal of small tumors. Additionally, this method could provide timely feedback of surgical outcome and improve the CC0-1 resection rate. Furthermore, it also enabled the precise detection and removal of tumors larger than 3 mm, which may improve the efficacy of comprehensive therapy for peritoneal cancer by ensuring the absence of residual tumor or the maximum diameter of residual lesions less than 2.5 mm after surgery, thusimproving the survival and prognosis of patients.

In addition, the extension of operative time represents a relevant factor for postoperative complications. Thus, intraoperative real-time and rapid imaging, whichfacilitate rapid treatment decisions that shorten the detection time and enable a timely feedback of surgical outcomes, may in turn reduce the risk of postoperative complications. Figure 7B shows that in the current study, the process of fluorescence-guided laparotomy was uncomplicated with a significantly shorter detection time compared with the conventional treatment group. The mean detection time of the fluorescence group was 34 s whereas the detection time of the conventional group was 110.8 s, representing a statistically significant increase of 3.26-fold compared with the fluorescence group.

## DISCUSSION

Integrin α_v_β_3_is widely expressed in endothelial cells on the surface and in the blood vessels of tumors but is rarely expressed in normal tissues. The expression of α_v_β_3_ in malignant tumors, such as melanoma [29], breast cancer [30], and ovarian cancer is significantly higher compared with normal tissues, and is closely associated with malignant behaviors including tumor cell proliferation, metastasis, and invasion. In particular, the results of a large-scale study on the expression of α_v_β_3_ in gastric cancer tissues conducted by Boger et al.indicated that α_v_β_3_ is widely expressed in gastric cancer tissues; thus, integrin α_v_β_3_ has become a new therapeutic target of gastric cancer [27]. Furthermore, integrins overall have gained increasing attention as therapeutic targets of tumors, especially α_v_β_3_ and α_v_β_5_. In turn, integrin receptors such as Cilengitide, an RGD cyclic peptide, monoclonal antibodies, and RGD peptides specifically bind to tumor tissues. The malevolent behaviors mediated by the resultant integrin antagonism during the occurrence and development of malignant tumors inhibittumor proliferation and promotes apoptosis. Currently, these receptors have entered Phase I clinical trials and have achieved good diagnostic and therapeutic outcomes in certain tumor cases.

The binding of integrin αvβ3 receptor to RGD is closely associated with its conformation and rigidity, working concentration, and ratios. Studies have confirmed that cyclic RGD dimers exhibit a higher targeting ability than that of the cyclic and linear RGD monomers and that the optimum αvβ3receptor-to-RGD binding ratio is 1:2 [31]. The study conducted by Cao et al. was consistent with these findings [32]. Hence, in the current study we have designed and synthesized a cyclic RGD and further improved the binding domain and affinity of RGD and αvβ3through modifications of ICG, so that each ICG molecule bound to two RGD molecules to yield the structural conformation ratio 1:2. Such RGD peptide sequence-containing probescan therefore be used to target tumor tissues that specifically express integrins including α_v_β_3_, to achieve precise positioning and consequenttreatment of tumors by using molecular imaging techniques.

Here, we verified the targeting ability of RGD-ICG by utilizing SGC-7901 cells as the experimental group and GES-1 cells as the control group. The fluorescent dye ICG can only be phagocytosed and retained in tumor cells by specific binding to the integrin α_v_β_3_ on the surface of cellular membranes via RGD, whereupon it can be revealed by imaging under near infrared light irradiation. In contrast, pure RGD and ICG cannot be used for targeted molecular imaging, as tissues cannot uptake the fluorescent substances. Additionally, the targeting ability of RGD-ICG is associated with the expression level of its receptors on cellular membranes. Therefore, it is possible to reveal a clear image of tumor tissues through the high differential in fluorescence expression between tumor and non-tumor cells owing to the high integrin expression in the latter. Additionally, the specificity of RGD in targeting tumor tissues has been verified in other studies [33, 34]. Furthermore, ICG represents an FDA-approved clinical drug and RGD is a polypeptide. Accordingly, in toxicity assessments the viability of tissues and cells was not significantly reduced by the increasing concentration of probes, indicating that the probe exhibited good histocompatibility.

The investigation of probe metabolism identified that the probe rapidly spread throughout the body after intravenous tail injection and started to accumulate in tumors and the liver after 30 min with a retention time up to 72 h, followed by excretion via the liver and digestive tract. Notably, the peritoneal tumor could be revealed by imaging between 24- to 72-h post-injection owing to the excretion of excessive probes from the body after 24 h, which enhanced the signal-to-noise ratio up to 4. The *in vivo* metabolism of RGD-ICG was similar to that observed in the study by Ito et al. [35], wherein epidermal growth factor receptor-ICG and carcinoembryonic antigen-ICG were synthesized for the diagnosis of peritoneal carcinomatosis from gastric cancer and for which it was found that the probe could be retained in tumors for approximately 7 days whereas excessive probes were excreted from the body within 24 h. The metabolism of the RGD-ICG probe was thus consistent with that of ICGalone [36, 37], which is rapidly excreted from the body and thereby can be used for tumor diagnosis.

In clinical practice, the selection of suitable patients for cytoreduction surgery and hyperthermic intraperitoneal chemotherapy of peritoneal carcinomatosis from gastric cancer significantly improves the survival, prognosis, and quality of life of patients. Currently, the selection of suitable patients mainly relies on preoperative and intraoperative staging that includes clinical signs and symptoms as well as physical and laboratory examinations. In general, patients with few symptoms also show few positive signs in physical examinations in the early stage of peritoneal carcinomatosis from gastric cancer. Hence, abdominal ultrasound, enhanced CT, and PET have become important preoperative assessments for this disease. However, it is difficult for these auxiliary examinations, including PET, to detect small lesions, especially tumor nodules less than 5 mm, which require further intraoperative assessment such as by laparoscopy and cytological examination of exfoliated cells [38]. In addition, PCI is key indicator in assessing the tumor burden of peritoneal carcinomatosis from gastric cancer that can effectively guide the selection of patients and predict surgical efficacy and CCR is also an important indicator in determining surgical efficacy and prognosis.

In this study, we carried out the diagnosis and treatment of peritoneal carcinomatosis from gastric cancer by using the RGD-ICG-based optical diagnostic system,which achieved 93.9% sensitivity and 100% specificity with a diagnostic accuracy rate of 93.9% and DI of 193.9%. This system has a high diagnostic performance that improves the intraoperative PCI and CCR indices and provides a timely feedback of surgical outcomes. In addition, it can reduce unnecessary removal and damage to normal tissue, as well as minimizing the risk of postoperative complications. In comparison, Harlaar et al. investigated the application of the IntegriSense 680-based optical diagnostic system for the diagnosis and treatment of ovarian cancer via the construction of a nude mouse model of ovarian cancer. Their study revealed that the diagnostic system exhibited a high diagnostic performance with 100% sensitivity, 88% specificity, DI of 188%, and diagnostic accuracy rate of 88% [39]. Sheth et al. also constructed a mouse model of ovarian cancer to investigate the diagnostic performance of the ProSense750-based optical diagnostic device in the diagnosis and treatment of ovarian cancer, demonstrating that it could achieve 100% sensitivity and 88% specificity [40]. Furthermore, Alexander et al. evaluated the diagnostic performance of the hGSA-NMP1-based optical diagnostic system on the nude mouse model of ovarian cancer and showed that this system achieved 91% sensitivity and 92% specificity in ovarian cancer [41]. Alternatively, Kondo et al. studied the diagnostic performance of the 5-ALA-based optical diagnostic system in the treatment of peritoneal carcinomatosis from colon cancer through the establishment of a nude mouse model of this disorder, finding that the system produced up to 100% sensitivity [42].

Together, these studies demonstrate that optical molecular imaging systems are able to achieve a high diagnostic performance in the treatment of malignant peritoneal cancer, including peritoneal carcinomatosis from gastric cancer. In addition, the RGD-ICG-based optical diagnostic system used in the current study could precisely detect tumor nodules with sizes of 1–2 mm, which effectively ensures a CCR^0-1^ resection rate during peritoneal cytoreduction surgery and improves the surgical efficacy. Furthermore, this system could perform real-time objective imaging that would be expected to reduce bias in treatment efficacy owing to differences in physician experience, ensure surgical efficacy, and might even reduce the risk of postoperative complications. Notably, the RGD-ICG-based optical diagnostic system could significantly reduce the detection time during laparotomy and facilitate rapid treatment decisions. Thus, to a certain extent, the use of this system shortened the operative time and thereby likely further reduced the risk of postoperative complications.

## MATERIALS AND METHODS

### Cell lines

The cell lines SGC-7901, GES-1, MDA-MB-231, MCF-7, BGC-823, and MGC-803 were purchased from Beijing China Infrastructure of Cell Line Resources and used in our study. Cells were cultured in RPMI 1640 supplemented with 10% FBS (HyClone,Thermo Scientific, Waltham, MA, USA), 1% penicillin, and 1% streptomycin at 37°C in a humidified atmosphere containing 5% CO_2_.

### Detection of integrin α_v_β_3_ expression in the cell lines by western blotting

Whole cell lysates were electrophoresed on SDS-PAGE and transferred to nitrocellulose membranes. Membranes were blotted with an anti-CD61 antibody (ab197662, Abcam, Cambridge, MA, USA) at the recommended concentration (1:500) overnight at 4°C and the bound primary antibodies were detected using peroxidase-conjugated secondary antibodies. Blots were developed using a SuperSignal enhanced chemiluminescence kit (Pierce, Rockford, IL, USA) and imaged on a Kodak Imager (Rochester, NY, USA). Proteins were analyzed by SDS-PAGE and transferred to Immobilon membranes. Blocking was performed by incubating the membranes with TBS, pH 7.4, with 0.05% Tween (TBS-T), containing 5% nonfat dry milk. Membranes were incubated with primary antibodies for 16 h at 4°C under continuous agitation, washed 3 times with TBS-T, and incubated with secondary antibodies for 1 h at room temperature. Detection of immunoreactive bands was performed using the enhanced chemiluminescence detection kit (Pierce). The protein levels that corresponded to the immunoreactive bands were quantified using ImagePC image analysis software (Scion Corp., Frederick, MD, USA).

### *In vitro* probe experiments

To assess cell viability, SGC-7901 and GES-1 cells grown until the logarithmic phase were harvested. The concentration of the cell suspension was adjusted and cells were inoculated into 96-well plates at the cell density of 10,000 cells/well. Then, 100-μL minimal medium supplemented with 10% FBS was aliquoted into each well while the marginal wells were provided with sterile PBS. The inoculated cells were incubated overnight at 37°C with 5% CO_2_ until the cell monolayers at the bottom of wells reachedapproximately 60–70% confluence. Subsequently, 100 μL medium supplemented with different concentrations of RGD-ICG (0, 25, 50, 100, 150, 200, 250, 300, 350, and 400 μg/ml) were added into 3 replicate wells while wells provided with RGD-ICG-free medium (0 mg/ml) were taken as the control group; The inoculated cells were incubated at 37°C with 5% CO_2_ for 24 h prior to observation under a microscope; 20uL3-(4,5-dimethylthiazol-2)- 2,5-diphenyl-tetrazolium bromide was added into each well followed by further incubation for 4 h; then, the medium in each well was carefully aspirated to terminate the cell culture and 150 μl dimethyl sulfoxide was added into each well followed by low-speed oscillation in a shaker for 10 min to fully dissolve the formazan crystals; The absorbance value of each well was measured at OD^490 nm^ by using an ELISA microplate reader (Multiskan, Thermo Fisher); The viability of cells was calculated according to the absorbance value by using the following formula:

cell viability = (Control OD − Experimental OD)/Control OD × 100%.

To detect the fluorescence generated by the RGD-ICG probe and its components, SGC-7901 and GES-1 cells grown until logarithmic phase were harvested. The concentration of the cell suspension was adjusted and cells were inoculated into 96-well plates at the cell density of 10,000 cells/well. Then, 200 μl minimal medium supplemented with 10% FBS was aliquoted into each well whereas the marginal wells were provided with sterile PBS. Each cell type contained four groups and each group comprised five replicate wells. The inoculated cells were incubated overnight at 37°C with 5% CO_2_ until the cell monolayers at the bottom of wells reached approximately 40−50% confluence.After washing the calls with PBS three times, 200 μl medium supplemented with different ingredients was added into the replicate wells. The control group received basic DMEM; the RGD group was supplemented with free RGD(100 nM),the ICG group received free ICG(100 nM), and the RGD-ICG group medium contained the probe (RGD-ICG) at 100 nM. The inoculated cells were incubated at 37°C with 5% CO_2_ for 30 min. The cell culture was then terminatedand the cells were carefully washed several times with PBS; subsequently, 100 μl basic DMEM culture medium was added into the well.A black plate was then placed in IVIS (LB983 NC100, Berthold, Germany) and the fluorescence signal of each cell was tested with the following parameters: fluorescence and excitation wavelength of 780 nm, filter wavelength of 845 nm, and exposure time 2000 ms.From the ROI curve the whole fluorescence signal value was calculated and the light intensity was normalized to each photon number per second per square centimeter per radian Angle (p/SEC/cm^2^/sr).We also incubate the probe of RGD-ICG(100 nM)with SGC-7901 and GES-1 as the same protocols mentioned above,captured images of these two types of cells under a fluorescence microscope.

### Establishment of murine gastric tumor models

All animals were purchased from the Department of Experimental Animals, Academy of Military Medical Sciences,Beijing,china. All experimental protocols were approved by the Institutional Animal Care and Use Committee (IACUC) at Tsinghua University and all the methods were carried out in accordance with the approved guidelines.

### Establish the subcutaneous tumors model from gastric cancer and capture dynamic imaging statusinthe model of subcutaneous tumors

Cells from the gastric cancer cell line, SGC-790l, were grown until logarithmic phase, harvested, the cell density measured, and a total of 1 × 10^7^ cells were dissolved in 200 μl PBS; The experimental nude mice were placed in an anesthetic chamber containing a 2% isoflurane/oxygen mixture. The anesthetized nude mice were subjected to a slow intraperitoneal injection of 200 μl PBS containing 1 × 10^7^ gastric cancer cell in right flank area.

After the tumor reach about 5 mm in the diameter, we examined the dynamic imaging status in the model of subcutaneous tumors.The model nude mice were randomly divided into two groups (*n* = 3). Then inject RGD-ICG and ICG separatelyby tail vein.The micewere then placed in IVIS (LB983 NC100, Berthold, Germany) and the fluorescence signal of subcutaneous tumor was tested with the following parameters: fluorescence and excitation wavelength of 780 nm, filter wavelength of 845 nm, and exposure time 2000 ms at the time points of 0 min, 30 min, 12 h, 24 h, 48 h, 72 h and 96 h. From the ROI curve the whole fluorescence signal value was calculated and the light intensity was normalized to each photon number per second per square centimeter per radian Angle (p/SEC/cm^2^/sr).

### Experimental methods for image-guided surgery

Establish the subcutaneous tumor model and recorded their maximum diameters by using calipers at day 3, 6, and 9 after injection with gastric cancer cell lines, SGC-7901. The imaging status was also collected at day 3, 6, and 9 after subcutaneous implantation with the gastric cancer cell line, SGC-7901. The imaging data of each time point, imposed under the same fluorescence signal intensity scale, were analyzed

The navigated surgery process to resect subcutaneous tumors from gastric cancer was as follows: the mice were subjected to intravenous tail injection of RGD-ICG molecular probe 24 h prior to the surgery. After setting the parameters of the fluorescence molecular imaging navigation system, the anesthetized experimental nude mice were placed on the surgical navigation system platform to detect the fluorescence signal in the body of the experimental nude mice. Then, tissues with greatest fluorescence signal were identified for complete resection and the excised tissue samples were fixed in formaldehyde solution followed by pathological H&E staining to confirm the nature of the tissue.

### Establishment and selection of a model of peritoneal carcinomatosis from gastric cancer

For the preparation and grouping of nude mice, 4-week-old female BALB/c (nu/nu) nude mice purchased from the Academy of Military Medical Sciences were housed in clean breeding cages and fed with low-fluorescence diets. The mice were randomly divided based on body weight into pre-injection, 3rd, 6th, 9th, and 12th day post-injection groups of 3 mice each. The nude mice were subjected to subsequent experiments after being adapted to the environment.

Cells from the gastric cancer cell line, SGC-790l, were grown until logarithmic phase, harvested, the cell density measured, and a total of 1 × 10^7^ cells were dissolved in 200 μl PBS; The experimental nude mice were placed in an anesthetic chamber containing a 2% isoflurane/oxygen mixture. The anesthetized nude mice were subjected to a slow intraperitoneal injection of 200 μl PBS containing 1 × 10^7^ gastric cancer cell at approximately 1 mm below the xiphoid. After the injection, the abdomen of the nude mice was gently pressed and the nude mice were returned to clean breeding cages after recovering from anesthesia.Prior to the injection and at day 3, 6, 9, and 12 after the injection, representative nude mice were sacrificed according to the experimental design and subjected to laparotomy in order to observe the formation of peritoneal tumors of the gastric cancer cell line, SGC-7901. The general condition of the peritoneal tumors, such as distribution, coloration, and hardness, was recorded. Then, three tumors were randomly selected and fixed in formaldehyde solution, followed by embedding in paraffin. The paraffin-embedded tissues were subjected to H&E staining and immunohistochemical staining in order to determine the expression of α_v_β_3_ to select the appropriate model of peritoneal carcinomatosis from gastric cancer for subsequent examination.

### Navigated cytoreduction surgery of peritoneal carcinomatosis from gastric cancer

For the preparation and grouping of nude mice, 4-week-old female nude mice were housed in clean breeding cages and fed with low-fluorescence diets. The mice were randomly divided into fluorescence and conventional groups of 12 nude mice each. The physicians were also divided into the two corresponding groups, in which physicians of the fluorescence group performed fluorescence-navigated laparotomy and assessment of tumor properties,whereas physicians of the conventional group performed laparotomy and assessment of tumor properties based on visualization and palpation. The diagnostic criteria of visualization for peritoneal carcinomatosis from gastric cancer includedvisible gray and rigid nodules distributed on the surface of intraperitoneal organs (including the large and small omentum, and mesentery). Experimental programs included the range, content, and time of laparotomy. The range of laparotomy included the entire intraperitoneal cavity, including the large and small omentum, mesentery, surfaces of peritoneal organs, pelvic organs, and parietal peritoneum.The content of laparotomy included the examination of all nodular masses within the range of the laparotomy to determine the nature of the tumors. In the fluorescence group, a positive nodule was defined as a fluorescently imaged nodule whereas a negative nodule was defined as a non-imaging nodule. In the conventional group, a positive nodule needed to be confirmed by 2 physicians based on the diagnostic criteria and assessment. Acontroversial positive lesion needed to be agreed to be classified as a positive nodule by both physicians after discussion whereas a negative lesion was confirmed by 2 physicians based on the diagnostic criteria and assessment. A controversial negative lesion needed to be agreed as classified as a negative lesion by both physicians after discussion. The time of laparotomy was assessed as the time required for the range and content of laparotomy to be ascertained.

For the surgical procedure in the fluorescence group, experimental nude mice were subjected to intravenous tail injection of 100 μl of 0.1 mg/ml RGD-ICG probe 24 h prior to surgery. b) The effort was divided between the 2 physicians in order to ensure a smooth experimental course. One of the physicians carried out the visualization and marked the imaging nodules while another physician operated the imaging devices. The experimental timing was initiated following the abdominal incisions of anesthetized nude mice.The white light was turned off prior to fluorescence-navigated laparotomy. Imaging nodules were marked based on the imaging results and the timing was stopped after the physicians were satisfied with the examination results. The maximum diameters of imaging tumors were measured using a caliper and all imaging and non-imaging nodules were excised. The tissues were fixed and preserved in formaldehyde solution for further processing. Both physicians examined the intraperitoneal cavity of the nude mice to ensure that there were no residual nodules.

For the surgical procedure in the conventional group, the anesthetized nude mice were examined after the abdominal incision. The experimental timing began when both physicians were prepared and initiated the laparotomy; for this group, the experiment was performedunder white light. Positive nodules were marked and the nature of the nodules within the predetermined range was examined. The timing was stopped when both physicians were satisfied with the results of laparotomy. Both nodules marked positive unmarked negative nodules were surgically removed, counted, and fixed in formaldehyde solution for preservation until further processing. Both physicians re-examined the nude mice to ensure that there were no residual nodules.

All removed peritoneal nodules were sent for pathological examination to further confirm the nature of the masses. The diagnostic performance of fluorescence navigation technology in this study, such as sensitivity, specificity, DI, and accuracy rate were assessed according to the pathological results.

### Statistical analysis

During the examination of excised tissues, a true positive (TP) was defined as a nodule detected by imaging and pathologically diagnosed as a tumor; a false positive (FP) was defined as a nodule detected by imaging but for which pathological examination did not find any tumor tissue; true negative (TN) referred to negative nodules with respect to both imaging and pathological examination; and false negative (FN) referred to negative nodules in imaging revealed by pathological examination as tumor tissue. Performance characteristics were calculated as follows: sensitivity = TP/TP + FN; specificity = TN/TN + FP;DI = sensitivity + specificity; Youden index = sensitivity + specificity − 1.

In this study, the statistical data was processed using SPSS19.0 software (Chicago, IL, USA). The measurement data are expressed as the means ± SD and were compared by using the *t-test* whereas the comparison among multiple groups utilized one-way analysis of variance (ANOVA). *P* < 0.05 was considered statistically significant.

## CONCLUSIONS

In summary, we showed that the RGD-ICG-based optical diagnostic system has a high diagnostic performance and reduced operative time, and provides real-time objective imaging in the treatment of peritoneal carcinomatosis from gastric cancer invivo. These featureswould be expected to facilitate the selection of suitable patients, improve the CCR^0-1^ resection rate for the quality of cytoreduction surgery,and reduce the risk of postoperative complications upon clinical application. Consequently, this system may eventually increase the surgical quality and improve the prognosis and quality of life of the patients.Our results thereby provide a rigid foundation for the future application of the RGD-ICG-based optical diagnosis system for the diagnosis and treatment of peritoneal carcinomatosis from gastric cancer.

## Abbreviations

RGD: Arg-Gly-Asp
CCR: completeness of cytoreduction
CT: computerized tomography
DI: diagnostic index
DMEM: Dulbecco's modified Eagle's medium
FN: false negative
FP: false positive
ICG: indocyanine green
PCI: peritoneal carcinoma index
PET: positron emission tomography
TN: true negative
TP: true positive.
